# Supplementary material for: Evaluation of the measurement properties of intimate partner violence screening instruments for the general population: A COSMIN-based international systematic review
Source: PLoS One. 2024 Nov 14;19(11):e0310297. doi: 10.1371/journal.pone.0310297 (PMC11563433; doi:10.1371/journal.pone.0310297)
Supplement: S2 File — (DOCX) [file pone.0310297.s002.docx]

**Search Strategies**

**PubMed:**

#1: (Intimate partner violence OR Spouse Abuse[MeSH Terms]) OR (Spouse Abuse[Title/Abstract] OR Intimate partner violence[Title/Abstract] OR IPV[Title/Abstract] OR Partner Violence, Intimate[Title/Abstract] OR Violence, Intimate Partner[Title/Abstract] OR Intimate Partner Abuse[Title/Abstract] OR Abuse, Intimate Partner[Title/Abstract] OR Partner Abuse, Intimate[Title/Abstract] OR Dating Violence[Title/Abstract] OR Violence, Dating[Title/Abstract]) :**[21223]**

#2: (Mass Screening OR Multiphasic Screening[MeSH Terms]) OR (((Screening instrument*[Title/Abstract] OR Screening tool*[Title/Abstract]) OR (Mass Screening*[Title/Abstract] OR Screening*, Mass[Title/Abstract] OR Screening*[Title/Abstract])) OR (Multiphasic Screening*[Title/Abstract] OR Screening*, Multiphasic[Title/Abstract] OR Automated Multiphasic Health Testing[Title/Abstract])) OR (Screened[Title/Abstract] OR detect[Title/Abstract] OR detected[Title/Abstract] OR detection[Title/Abstract]): **[3314877]**

#1 AND #2: **[2927]**

**WOS:**

#1: TS=(Spouse Abuse OR Intimate partner violence OR IPV OR Partner Violence, Intimate OR Violence, Intimate Partner OR Intimate Partner Abuse OR Abuse, Intimate Partner OR Partner Abuse, Intimate OR Dating Violence OR Violence, Dating): **[46250]**

#2: TS=(Screening instrument* OR Screening tool* OR Mass Screening* OR Screening*, Mass OR Screening* OR Multiphasic Screening* OR Screening*, Multiphasic OR Automated Multiphasic Health Testing OR Screened OR detect OR detected OR detection): **[13645409]**

#1 AND #2 : **[6103]**

**PQDT:**

(Spouse Abuse OR Intimate partner violence OR IPV OR Partner Violence, Intimate OR Violence, Intimate Partner OR Intimate Partner Abuse OR Abuse, Intimate Partner OR Partner Abuse, Intimate OR Dating Violence OR Violence, Dating) AND (Screening instrument* OR Screening tool* OR Mass Screening* OR Screening*, Mass OR Screening* OR Multiphasic Screening* OR Screening*, Multiphasic OR Automated Multiphasic Health Testing OR Screened OR detect OR detected OR detection)

**ALL Abstract & summary text: [285]**

**EBSCO:**

(Spouse Abuse OR Intimate partner violence OR IPV OR Partner Violence, Intimate OR Violence, Intimate Partner OR Intimate Partner Abuse OR Abuse, Intimate Partner OR Partner Abuse, Intimate OR Dating Violence OR Violence, Dating) AND (Screening instrument* OR Screening tool* OR Mass Screening* OR Screening*, Mass OR Screening* OR Multiphasic Screening* OR Screening*, Multiphasic OR Automated Multiphasic Health Testing OR Screened OR detect OR detected OR detection)

**AB Abstract and Author Supplied abstract: [304]**

**EMBASE:**

(Spouse Abuse OR Intimate partner violence OR IPV OR Partner Violence, Intimate OR Violence, Intimate Partner OR Intimate Partner Abuse OR Abuse, Intimate Partner OR Partner Abuse, Intimate OR Dating Violence OR Violence, Dating) AND (Screening instrument* OR Screening tool* OR Mass Screening* OR Screening*, Mass OR Screening* OR Multiphasic Screening* OR Screening*, Multiphasic OR Automated Multiphasic Health Testing OR Screened OR detect OR detected OR detection)

**Title and abstract: [100]**
