# Supplementary material for: Evaluation of the measurement properties of intimate partner violence screening instruments for the general population: A COSMIN-based international systematic review
Source: PLoS One. 2024 Nov 14;19(11):e0310297. doi: 10.1371/journal.pone.0310297 (PMC11563433; doi:10.1371/journal.pone.0310297)
Supplement: S1 Table — (DOCX) [file pone.0310297.s003.docx]

S1 Table 1 The extraction process information

| Author, year | Reviewers | The third researcher | Included or not | Data extractors | The third researcher | Date | |
| --- | --- | --- | --- | --- | --- | --- | --- |
|  |  |  |  |  |  | Y.L. | G.W. |
| Harriet 2006 | Y.L. and G.W. | S.O. (All different opinions between the researchers were resolved under the help of the third researcher.) | Included | Y.L. and G.W. | J.C. (The third researcher would examine the extracted data and address any differences encountered.) | 2023-10-25 | 2023-11-02 |
| Kim 1997 | Y.L. and G.W. |  | Included | Y.L. and G.W. |  | 2023-10-25 | 2023-11-02 |
| Trevor 2005 | Y.L. and G.W. |  | Included | Y.L. and G.W. |  | 2023-10-25 | 2023-11-02 |
| Nuberg 2008 | Y.L. and G.W. |  | Included | Y.L. and G.W. |  | 2023-10-27 | 2023-11-03 |
| Maria 2014 | Y.L. and G.W. |  | Included | Y.L. and G.W. |  | 2023-10-28 | 2023-11-03 |
| Claudia 2002 | Y.L. and G.W. |  | Included | Y.L. and G.W. |  | 2023-10-28 | 2023-11-04 |
| Helen 2015 | Y.L. and G.W. |  | Included | Y.L. and G.W. |  | 2023-10-28 | 2023-11-04 |
| Trevor 2005 | Y.L. and G.W. |  | Included | Y.L. and G.W. |  | 2023-10-28 | 2023-11-06 |
| Leila 2006 | Y.L. and G.W. |  | Included | Y.L. and G.W. |  | 2023-10-30 | 2023-11-06 |
| KATARINA 2007 | Y.L. and G.W. |  | Included | Y.L. and G.W. |  | 2023-11-01 | 2023-11-08 |
| Zink 2007 | Y.L. and G.W. |  | Included | Y.L. and G.W. |  | 2023-11-02 | 2023-11-08 |
| Wirtz 2016 | Y.L. and G.W. |  | Included | Y.L. and G.W. |  | 2023-11-02 | 2023-11-08 |
| Alexander 2016 | Y.L. and G.W. |  | Included | Y.L. and G.W. |  | 2023-11-02 | 2023-11-10 |
| Shan Shan He 2013 | Y.L. and G.W. |  | Included | Y.L. and G.W. |  | 2023-11-03 | 2023-11-10 |
| Guilherme 2021 | Y.L. and G.W. |  | Included | Y.L. and G.W. |  | 2023-11-03 | 2023-11-11 |
| Lenore 2021 | Y.L. and G.W. |  | Included | Y.L. and G.W. |  | 2023-11-04 | 2023-11-11 |
| Taghi 2016 | Y.L. and G.W. |  | Included | Y.L. and G.W. |  | 2023-11-05 | 2023-11-11 |
| Young-Ju 2017 | Y.L. and G.W. |  | Included | Y.L. and G.W. |  | 2023-11-05 | 2023-11-12 |
| Sahar 2022 | Y.L. and G.W. |  | Included | Y.L. and G.W. |  | 2023-11-06 | 2023-11-12 |
| Hardip 2007 | Y.L. and G.W. |  | Included | Y.L. and G.W. |  | 2023-11-06 | 2023-11-14 |
| Linda 2011 | Y.L. and G.W. |  | Included | Y.L. and G.W. |  | 2023-11-07 | 2023-11-14 |
| Rita 2018 | Y.L. and G.W. |  | Included | Y.L. and G.W. |  | 2023-11-08 | 2023-11-15 |
| Maria 2022 | Y.L. and G.W. |  | Included | Y.L. and G.W. |  | 2023-11-09 | 2023-11-16 |
| Victoria 2021 | Y.L. and G.W. |  | Included | Y.L. and G.W. |  | 2023-11-10 | 2023-11-16 |
