# Supplementary material for: Evaluation of the measurement properties of intimate partner violence screening instruments for the general population: A COSMIN-based international systematic review
Source: PLoS One. 2024 Nov 14;19(11):e0310297. doi: 10.1371/journal.pone.0310297 (PMC11563433; doi:10.1371/journal.pone.0310297)
Supplement: S2 Table — (DOCX) [file pone.0310297.s004.docx]

S2 Table 2 Boxes of COSMIN Risk of Bias Checklist of the Included Studies

| Included studies  (Author, year) | Measurement properties | | | | | | | | | | Included or not |
| --- | --- | --- | --- | --- | --- | --- | --- | --- | --- | --- | --- |
|  | Content validity | | Internal structure | | | Remaining measurement properties | | | | |  |
|  | PROM development | Content validity | Structural validity | Internal consistency | Cross-culture validity | Reliability | Measurement error | Criterion validity | Hypotheses testing | Responsiveness |  |
| Harriet 2006 |  |  |  |  |  |  |  | ✓ |  |  | Included |
| Kim 1997 | ✓ |  |  |  |  |  |  | ✓ |  |  | Included |
| Trevor 2005 |  |  |  |  |  |  |  | ✓ |  |  | Included |
| Nuberg 2008 | ✓ |  |  |  |  |  |  | ✓ |  |  | Included |
| Maria 2014 | ✓ |  | ✓ | ✓ |  |  |  |  |  |  | Included |
| Claudia 2002 | ✓ |  |  | ✓ |  | ✓ |  |  |  |  | Included |
| Helen 2015 |  |  |  | ✓ |  |  |  |  |  |  | Included |
| Trevor 2005 |  |  |  |  |  |  |  | ✓ |  |  | Included |
| Leila 2006 | ✓ |  |  | ✓ |  |  |  |  |  |  | Included |
| KATARINA 2007 | ✓ |  |  |  |  |  |  | ✓ |  |  | Included |
| Zink 2007 | ✓ |  |  | ✓ |  |  |  | ✓ |  |  | Included |
| Wirtz 2016 | ✓ |  |  | ✓ | ✓ |  |  |  |  |  | Included |
| Alexander 2016 | ✓ |  |  | ✓ | ✓ |  |  |  |  |  | Included |
| Shan Shan He 2013 | ✓ |  |  | ✓ |  |  |  |  |  |  | Included |
| Guilherme 2021 | ✓ |  | ✓ | ✓ |  |  |  |  | ✓ |  | Included |
| Lenore 2021 | ✓ |  |  | ✓ |  |  |  | ✓ |  |  | Included |
| Taghi 2016 | ✓ | ✓ | ✓ | ✓ |  | ✓ |  |  |  |  | Included |
| Young-Ju 2017 | ✓ |  |  |  |  |  |  | ✓ |  |  | Included |
| Sahar 2022 | ✓ | ✓ | ✓ | ✓ |  | ✓ |  |  |  |  | Included |
| Hardip 2007 | ✓ |  |  |  |  |  |  | ✓ |  |  | Included |
| Linda 2011 | ✓ | ✓ |  | ✓ |  |  |  |  |  |  | Included |
| Rita 2018 | ✓ |  | ✓ | ✓ |  | ✓ |  | ✓ |  |  | Included |
| Maria 2022 | ✓ | ✓ | ✓ | ✓ |  | ✓ |  |  |  |  | Included |
| Victoria 2021 | ✓ |  |  | ✓ |  |  |  |  |  |  | Included |
