# Supplementary material for: Evaluation of the measurement properties of intimate partner violence screening instruments for the general population: A COSMIN-based international systematic review
Source: PLoS One. 2024 Nov 14;19(11):e0310297. doi: 10.1371/journal.pone.0310297 (PMC11563433; doi:10.1371/journal.pone.0310297)
Supplement: Attachment — Submitted filename: Response to Reviewers.docx [file pone.0310297.s006.docx]

Dear Editor-in-Chief and reviewers，

Thank you for the quick and positive feedback for the manuscript. We sincerely thank you very much for your constructive comments and suggestions on further revisions to improve the quality of this manuscript. Appended to this letter is our point-by-point responses to the comments and suggestions. Changes made in the manuscript are marked in red in the uploaded file. A clean and a track-change copies of the revised manuscript and supporting information files have been uploaded.

We hope that you find our responses satisfactory, and that the manuscript is now acceptable for publication in the PLOS ONE.

Yours sincerely,

Jiarui CHEN

Xiangya School of Nursing,

Central South University

**Reviewer 1:**

1. I believe that the topic is important, however, the authors did not make a strong enough case for why this article is important. Also, the word "best" should be avoided and replaced with a term more appropriate for academic works and audiences. Finally, was this an international/global review (I think it was), and if so, the authors should consider a thorough review of cultural factors related to screening instruments for IPV.

**Response:** Thank you very much for your comments. We have noticed that using “best” is not appropriate in our manuscript. The title revision is as follows: “Evaluation of the measurement properties of intimate partner violence screening instruments for the general population: a COSMIN-based international systematic review”. -Page 1 lines 1-2.

Moreover, for culture factors, we added corresponding contents in the part of results. The revision is as follows: “Out of the 23 included studies, a total of 18 IPV screening instruments were reported. The 18 instruments were mainly (17 instruments) original developed in English. Among them, M-PVS was translated from English to German, CTS-2 was translated into two languages, namely Italian and Portuguese, WCFSI was translated from English to Chinese, SCIRS-SF was translated from English into Portuguese, a single violence question was translated from Spanish to English, Haj-Yahia's questionnaire was translated from English into Persian, NORAQ was translated from English into Arabic, PMWI-SF was translated from English into Portuguese, and MSDV-2.0 was translated from English into Spanish. The other instruments used a single language (English or Persian) and tested in their target population. The 18 instruments’ target populations contained women or married women, couples, intimate/romantic/dating relationships, and community populations among general population.” -Page 11 to 12 lines 203-213.

We consider that the translated IPV screening instruments could be tested in corresponding participants different from their target population, which could proof this IPV screening instruments adopted their culture.

**Reviewer 2:**

1. This paper assumes that the reader knows a lot about psychometrics and COSMIN. Both topics are quite complex and especially as COSMIN is not that well known outside of psychometrics, it would be useful to provide the reader with more information to help them interpret the manuscript. The manuscript would benefit from a table or figure explaining concepts such as construct validity, internal consistency etc. and how COSMIN ideally wants these to be measured by studies and what is considered to be less adequate (e.g. that for reliability a study would receive a sufficient rating if the ICC or weighted Kappa ≥ 0.70, the study would be insufficient if the ICC or weighted Kappa was < 0.70 and would be indeterminate is neither ICC nor weighted Kappa was recorded).

**Response:** Thank you for your opinions. After your comments, we have noticed that we haven’t add the published protocol of this review as a reference. Actually, before we conducted the full review, we have published a protocol titled on “Intimate Partner Violence Screening Instruments: A Protocol for a COSMIN-Based Systematic Review” (DOI: 10.3390/ijerph20021541). Therefore, the methodology of our study was based on this protocol, the contents of these concepts such as construct validity, internal consistency etc. and how COSMIN ideally wants these to be measured by studies and what is less adequate were not repeated in our manuscript. We have added corresponding explanations in the method part as follows: “The methodology of the current study was based on our published protocol (DOI: 10.3390/ijerph20021541). The protocol (24) for this study was registered in the International Prospective Register of Systematic Reviews (PROSPERO; number CRD42022365247 (24)).” –Page 5 lines 98-100.

2. Abstract

Aim: don’t think need added part of, "for their users". Recommendations is fine.

PQDT and EBSCO PBSC should be in full, not the acronym.

**Response:** Thanks for your suggestions. The revisions are as follows: “Aim: To systematically appraise, compare, and summarize the measurement properties of intimate partner violence screening instruments for the general population and provide recommendations based on COSMIN guideline.

Methods: We searched PubMed, Embase, Web of Science, ProQuest Dissertations & Theses Global and EBSCO Psychology Behavioral Sciences Collection from their establishment to March 2024 using systematic search strategies.” -Page 2 lines 24-28.

3. Introduction

Line 46: What do you mean by other relationship types? I would assume it’s some form of romantic/dating or sexual relationship? This needs to be made clear.

**Response:** Thank you very much for your suggestions. According to your suggestions, we read again the literature. We apologized for the confusions. The revision is as follows: “ Intimate partners may include couples, ex-couples, boyfriends/girlfriends, dating partners, sexual partners, or other romantic type of relationship.” -Page 3 lines 46-47.

4. Line 47 – 49: The sentence about domestic violence isn’t as easy to understand as it could be. Suggest that you explain that you’re going to refer to the concept as intimate partner violence, rather than domestic violence as you’re only looking at instruments that address abuse between romantic/dating partners rather than abuse between family members (e.g. abuse of children or elderly relatives).

**Response:** Thank you for your comments, we apologized for the confusions. The revision is as follows: “The term “domestic violence” is a broader term that contains the abuse of children or the elderly or the abuse by any member from the family, while it was used in many countries to refer to intimate partner violence (5)” -Page 3 lines 48-50.

5. Line 52 – 57: When you say the physical health consequences of IPV are well documented and the mental health consequences are frequently documented, what do you mean? Are there differences between the amount of evidence available for physical and mental health? Are associations between IPV and physical health better established? Or are the links between IPV and physical and mental health equally well evidenced? The wording of this part of the manuscript implies a hierarchy of evidence, but I'm not sure if that's what you meant.

**Response:** Thanks for your comments. After group discussion, we recognized that you are right. The revision is as follows: In a study by the World Health Organization, 27% of women aged 15–49 years had been subjected to physical and/or sexual violence by their partners, and 13% of them had experienced such events in the previous 12-month period (7). IPV can result in negative physical and emotional outcomes for the people who experience violence and their families (2). The physical health consequences include acute illnesses and chronic and pain-based disorders, such as respiratory, urinary tract, and sexually transmitted illnesses, insomnia, headache, menstrual-related disorders, pelvic pain, and functional gastrointestinal and reproductive diseases (8-10). Mental health issues are also reported among people who are experiencing or have experienced IPV, and these can have potentially serious consequences. Anxiety, depression, posttraumatic stress disorder, and suicide are all common among this population (11, 12). Importantly, the severity of these mental health issues tends to escalate with the severity of the IPV experienced.” -Page 3 lines 52-61.

6. Line 63: "Because of the difficulty of meeting the cut-offs" – I’m not sure what you mean by this. This should be clearly explained.

**Response:** Thanks for your comments. We have added the explanation in the part of discussion in our manuscript, the added contents are as follows: “ The IPV screening instruments in our review had many response options (41, 45, 46), which means there is no unified standard with which to determine the severity of IPV or to measure the cut-off scores of IPV screening instruments. As we mentioned before, because of unwilling disclosing or concealing the experiences of IPV, the results of screening cannot meet the cut-off scores, leading to the prevalence of IPV would be underestimated. In future studies, we hope that researchers will establish a rigorous standard that considers the severity of IPV as well as consistent cut-off scores.” -Page 21 lines 332-337.

7. Line 89: I wouldn’t give the example of female patients. State that you include studies on IPV screening instruments targeting both males and females.

**Response:** Thanks for your comments. We have noticed our expression was described unclearly. The revisions are as follows: “In this study, we hope to explore the methodological properties of IPV screening instruments targeting general population in non-specific population. Therefore, we will include the studies on IPV screening instruments targeting both males and females.” -Page 4 lines 81-83.

8. Methods

Provide references for COSMIN.

COSMIN addresses the psychometric properties of measurement instruments. The risk of bias tool is an adaptation of the checklist and its four-point rating system into a version exclusively for use in systematic reviews of Patient Reported Outcome Measures and assesses risk of bias of studies on measurement properties – Mokkink et al (2018) COSMIN Risk of Bias checklist for systematic reviews of Patient-Reported Outcome Measures. Qual Life Res. It’s not specifically about IPV screening instruments, as could be interpreted here.

**Response:** Thank you very much for your suggestion. The revised content is as follows: “The COSMIN guideline (20) provides methodology on addressing the risk of bias in studies that aimed at instruments developing, rating their measurement properties, evaluating the overall quality of evidence for each measurement property, and providing recommendations based on the overall quality evidence. The protocol for the current study was registered in the International Prospective Register of Systematic Reviews (PROSPERO; number CRD42022365247).” -Page 5 lines 95-98.

9. Line 114/115 – why did you contact the author of each included study to review the search results? Were reference lists of review articles identiﬁed in the literature search checked for relevant studies?

**Response:** Thanks for your question. We apologized for the expression unclear. The revised content is as follows: “ The reference lists of review articles identiﬁed in the literature search were checked for relevant studies. ” -Page 5 line 108.

10. Inclusion/Exclusion criteria isn't detailed enough, use PICO (population, interventions, comparators and outcomes).

I would be more inclined to say people who are victims/survivors of IPV rather than people who were suffering from IPV.

Were there exclusions for language?

**Response:** Thanks for your suggestions. Because we don’t cite our published protocol in our manuscript, we apologized for the confusions. The contents on PICO in our protocol are as follows: “In this study, we adapted the ‘objectives’ section of PRISMA 2020 checklist by changing ‘Population(s), Interventions, Comparators and Outcomes’ into ‘Construct, Populations, Type of Instruments and Measurement Properties’. The main search concepts are the consequences of IPV (Construct), people who suffer from IPV (Population), PROMs (Type of instrument(s)), and measurement properties.”

Additionally, the questions for languages we wrote in the inclusion criteria, the revisions are as follows: “Therefore, we included primary studies that (1) reported screening IPV instruments designed for people in the general population who were victims of IPV, (2) described the processes of development and/or evaluation of one or more measurement properties for the eligible instrument(s), (3) had full-text availability, and (4) articles were published in English. According to the COSMIN guideline, we excluded original studies that used the IPV screening instruments only for outcomes measurements not evaluating measurement properties.” -Page 6 lines 112-116.

11. Study selection

Examples of the Joanna Briggs Institute SUMARI search filters used would be helpful for someone replicating the review.

**Response:** Thanks for your suggestions. We used the Joanna Briggs Institute SUMARI search filter in our study, which was helpful for us. We wrote in our manuscript; the content is as follows: “We then imported the articles into Joanna Briggs Institute SUMARI search filters to identify the studies with psychometric properties linked to terms related to IPV screening instruments.” -Page 7 lines 118-120.

12. Who reviewed the titles and abstracts?

**Response:** Thanks for your question. We have added the researchers who reviewed the titles and abstracts in our manuscripts, and the researchers who extracted data and conducted quality appraisal and data synthesis, the revisions are as follows: “ Two independent researchers (Q.X. and K.C.) undertook the first manual filtering of the articles’ titles and abstracts based on the eligibility criteria. The full-text articles not excluded in the second manual screening phase were read independently by two researchers (Y.L. and G.W.). All different opinions between the researchers were resolved under the help of the third researcher (S.O.).” -Page 7 lines 120-123.

“First, we customized the data extraction table following a discussion in the research group. Then, two researchers (Y.L. and G.W.) separately extracted the corresponding data based on the content of the data extraction table. Finally, the third researcher (J.C.) would examine the extracted data and address any differences encountered.” -Page 7 lines 125-127.

“Two researchers (Y.L. and G.W.) independently assessed the measurement properties of the IPV screening instruments using the COSMIN methodology. The third researcher (J.C.) handled any disagreements related to the process.” -Page 8 lines 143-145.

13. Data abstraction

This is more of a computer science term, I would title as data extraction.

PROM needs writing out in full the first time it’s mentioned.

**Response:** Thanks for your opinions. The title revision is as follows: “ Data extraction”-Page 7 line 124. Additionally, the first time we mentioned PROMs, we have written out in full in the introduction, the content is as follows: “ The main purpose of this systematic review was therefore to identify the current IPV screening instruments in use for the general population and to evaluate their measurement properties based on the COSMIN methodology for systematic reviews of patient-reported outcome measures (PROMs) (20, 21) and the COSMIN Risk of Bias checklist for systematic reviews of PROMs (22).” -Pages 4 to 5 lines 85-89.

14. Characteristics of the included studies

Were the instruments tested mainly with males or females? or a mixture of both?

**Response:** Thanks for your questions. We have noticed that our expression was unclear, the revision is as follows: “Six screening instruments were tested in women and men, and twelve screening instruments only tested in women.” -Page 12 lines 212-213.

15. Methodological quality and measurement property ratings

What were the psychometric properties that were reported the least in the studies?

**Response:** Thanks for your questions. We have added the content in our manuscript that is as follows: “The two least frequently reported measurement properties in the included studies were cross-culture validity and hypotheses testing. Two measurement properties (i.e., measurement error and responsiveness) were not assessed.” -Page 12 lines 222-224.

16. More information is needed to explain why the measurement properties were given the ratings that they were. What was presented in the studies for the internal consistency, reliability and cross-cultural validity to be rated as very good? What was missing for criterion validity to be rated inadequate?

**Response:** Thank you for your questions. Our study conducted based on COSMIN guideline, we firstly published our protocol, which gave all details on how to give the ratings on the measurement properties. Therefore, through group discussion, we decided that we would not repeat again in the part of method in this manuscript. In the part of discussion, we explained the detailed reason why occurred this situation, the part contents are as follows: “None of the studies adhered to the minimum requirement recommended by the COSMIN Risk of Bias checklist that 30 experts evaluate the content validity of an instrument (22). According to many systematic reviews based on COSMIN methods, it is common for content validity to be of a doubtful or inadequate methodological quality (47, 48). In our opinion, there are two reasons for this phenomenon. First, researchers may overlook the basic criteria and importance of evaluating the content validity of an instrument as per the COSMIN Risk of Bias checklist. Second, the minimum number of experts suggested in the COSMIN Risk of Bias checklist to assess content validity is difficult for researchers to achieve.” -Page 19 lines 289-295.

17. Should be clearer how many studies measured each psychometric property for each instrument, I assume with 18 instruments and 23 studies, most instruments will have only been assessed by one study, but this isn’t clear. A table, figure or some reporting would help with this. Readers should understand this before the discussion.

**Response:** Thank you very much for your opinions. We have noticed that our contents are unclear, therefore, we added a table in our manuscript. The table is as follows: (-Pages 10 to 11)

**Table 2 The number of included studies and instruments**

| Included instrument | | Author, Year |
| --- | --- | --- |
| full name | acronym |  |
| Partner Violence Screen | PVS | Harriet 2006 |
|  |  | Kim 1997 |
|  |  | Trevor 2005 |
| the modified version of Partner Violence Screen | M-PVS | Nuberg 2008 |
| the revised Conflict Tactics Scale | CTS-2 | Maria 2014  Claudia 2002  Helen 2015  Trevor 2005 |
| Abuse Assessment Tool | AAT | Leila 2006 |
| Abuse Screening Inventory | ASI | KATARINA 2007 |
| Abuse Behavior Inventory | ABI | Zink 2007 |
| the Assessment Screen to Identify Survivors Toolkit for Gender Based Violence | ASIST-GBV | Wirtz 2016  Alexander 2016 |
| Women’s Coerced First Sexual Intercourse | WCFSI | Shan Shan He 2013 |
| the short form of Sexual Coercion in Intimate Relationships Scale | SCIRS-SF | Guilherme 2021 |
| The South Asian Violence Screen | SAVS | Lenore 2021 |
| a screening instrument for domestic violence | a screen instrument for DV | Taghi 2016 |
| a single violence question | a single violence question | Young-Ju 2017 |
| Haj-Yahia's questionnaire | Haj-Yahia's questionnaire | Sahar 2022 |
| Humiliation-Afraid-Rape-Kick | HARK | Hardip 2007 |
| NorVold Domestic Abuse Questionnaire | NORAQ | Linda 2011 |
| the short form of Psychological Maltreatment of Women Inventory | PMWI-SF | Rita 2018 |
| the short version of Multidimensional Scale of Dating Violence | MSDV-2.0 | Maria 2022 |
| Intimate Partner Violence–Brief Self-Screener | IPV-BSS | Victoria 2021 |

18. Discussion

Some discussion of what evidence is currently missing that would elevate existing measures from a B to an A may be interesting, especially with regards to directions for future research.

**Response:** Thank you very much for your suggestions, we have added the details are as follows: “Through the above analysis, future studies on developing IPV screening instruments can consider the following points: 1) recognize the importance of content validity and 30 experts evaluate the content validity of an instrument, 2) use confirmatory factor analysis to test structural validity, 3) select an appropriate “gold standard” to calculate the correlations or the area under the receiver operating curve to reflect criteria validity, 4) compare the measurement properties between new IPV screening instruments and other existed IPV screening instruments, and 5) report all measurement properties as much as possible according to the COSMIN guideline, which will benefit the IPV screening instruments elevating from category “B” to “A”.” -Pages 20 to 21 lines 318-324.

19. Generally, the discussion section is very well written. My only comment is that the rest of the paper refers to IPV among both men and women, but the discussion only mentions women being reluctant to report. The discussion would benefit from some inclusion about the majority of instruments only being tested with women.

**Response:** Thank you for your comments. We have noticed that our discussions were not sufficient. The more details are as follows: “The IPV screening instruments in our review could be administered via self-report (45) or face-to-face interviews (28, 40). We had difficulty identifying which method would be better for screening IPV in the general population. Due to cultural factors, irrespective of whether self-report or face-to-face interviews are used, this population often does not actively disclose their experiences of IPV in their daily lives and even conceal the fact of experiencing IPV. It is noticeable that a cross-sectional study (49) among general population has suggested women reported higher levels of concerns and psychological distress than men. We therefore suggest that future studies using IPV screening instruments should combine self-report and face-to-face interviews to compare the prevalence of IPV among women and men in the general population.” -Page 21 lines 327-332.

20. **Table 2**

Table 2 may benefit from more detail about the thematic content of the instruments. For example, categories of questions. Alternatively, this could be a supplementary table.

**Table 2 and 3**

The acronym and full name of each instrument would be useful here, so the reader don't have to look back to the main text.

**Table 3**

Make it clear the levels of evidence is using the GRADE criteria.

There are spelling mistakes in the figure presented.

**Response:** Thank you very much for your comments. We adjusted the contents of the tables according to your suggestions; all updated tables are as follows: (-Pages 14 to 18)

**Table 3. Description of the Included IPV Screening Instruments**

| Instrument | Categories of Questions | Number of Items | Item generation | Mode of Administration | Response Options | Range of Scores |
| --- | --- | --- | --- | --- | --- | --- |
| PVS | ①+safety | 3 | the original version of PVS | self-report | yes or no | not applicable |
|  |  | 3 | not applicable |  | yes or no | not applicable |
|  |  | 3 | the original version of PVS |  | yes or no | not applicable |
| M-PVS | ① | 5 | the original version of PVS | interview | yes or no | not applicable |
| CTS-2 | ①②③④ | 78 | the original version of CTS | self-report | 8-point-likert-type | 78~624 |
|  | ①②③④ | 78 | the original version of CTS |  | 8-point-likert-type | 78~624 |
|  | ①③④ | 32 | the original version of CTS |  | 8-point-likert-type | 32~256 |
|  | ①②③ | 78 | the original version of CTS |  | 7-point-likert-type | 0~468 |
| AAT | ①②③④ | 67 | the finding of a qualitative study and literature review | self-report | 5-point-likert-type | 0~268 |
| ASI | ①②③ | 16 | the texted version of ASI | interview | 11-point-likert-type and yes or no | not applicable |
| ABI | ①③ | 29 | not applicable | self-report | 5-point-likert-type | 29~145 |
| ASIST-GBV | ①②③④ | 7 | the qualitive research and systematic review | self-report | yes or no | not applicable |
|  |  | 7 | the qualitive research and systematic review |  | yes or no | not applicable |
| WCFSI | ①②③④ | 33 | the original version of SCIRS | self-report | 6-point-likert-type | 0~215 |

①physical violence; ② sexual violence; ③emotional-psychological abuse; ④controlling behaviors

**Table 3. Description of the Included IPV Screening Instruments (Continued)**

| Instrument | Categories of Questions | Number of Items | Item generation | Mode of Administration | Response Options | Range of Scores |
| --- | --- | --- | --- | --- | --- | --- |
| SCIRS-SF | ② | 9 | the original version of SCIRS | self-report | 6-point-likert-type | 0~45 |
| SAVS | ①③ | 14 | literature review & the first author's over 20-year experience | self-report | 5-point-likert-type | 14~70 |
| a screen instrument for DV | ①②③ | 20 | HITS & VAWI | self-report | 5-point-likert-type | 0~80 |
| a single violence question | ① | 1 | The partner table | interview | yes or no | not applicable |
| Haj-Yahia's questionnaire | ①②③④ | 32 | CTS+PMWI+MWA+ISA+ABI | self-report | dichotomous scale | not applicable |
| HARK | ①②③④ | 4 | AAS | self-report | yes=1 & no=0 | 0~4 |
| NORAQ | ①②③ | 10 | the English version of NORAQ | self-report | yes or no | not applicable |
| PMWI-SF | ①②③ | 14 | the Portuguese version of PMWI | self-report | 5-point-likert-type | 14~70 |
| MSDV-2.0 | ①②③④ | 42 | the original version of MSDV and new items related to online violence and sexual violence | self-report | 5-point-likert-type | 42~210 |
| IPV-BSS | ①②③④ | 4 | WHO-IPV | self-report | yes or no or not applicable | not applicable |

①physical violence; ② sexual violence; ③emotional-psychological abuse; ④controlling behaviors

**Table 4. The language and Participants of the Included IPV Screening Instruments**

| Instrument | Original Language | Target Population | Country & Available Translated Version | Participants & Settings | N |
| --- | --- | --- | --- | --- | --- |
| PVS | English | all noncritical English-speaking women | Canada & English | English-speaking women & health care settings | 1602 |
|  |  |  | Denver & English | all noncritical English-speaking women & hospital-based ED | 322 |
|  |  |  | America & English | male victims of IPV & ED | 116 |
| M-PVS | English | women in different institutions from German-speaking countries | Basel & German | German-language women & psychiatric clinic settings | 115 |
| CTS-2 | English | couples in general population | Italy & Italian | non-abused women and victims of IPV & primary health care centers and women's shelters | 209 |
|  |  |  | Rio de Janeiro & Portuguese | pregnant women and premature childbirth & Brazilian context | 774 |
|  |  |  | Victoria & English | separated couples & Family mediation settings | 121 |
|  |  |  | America & English | male victims of IPV & ED | 116 |
| AAT | English | Jamaican women | Jamaica & English | Jamaican women & primary health care clinics and crisis centers | 205 |
| ASI | English | random female Swedish sample | Sweden & English | randomized sample of women & health care settings | 699 |
| ABI | English | English-speaking women | Ohio & English | White, African, and American Women & primary care waiting rooms | 392 |
| ASIST-GBV | English | refugees and internally displaced persons | Ethiopia and Colombia & English | female refugees and internally displaced persons & humanitarian settings | 503 |
|  |  |  | Ethiopia and Colombia & English |  | 998 |
| WCFSI | English | intimate relationships | China & Chinese | university students & heterosexual dating settings | 927 |

**Table 4. The language and Participants of the Included IPV Screening Instruments (Continued)**

| Instrument | Original Language | Target Population | Country & Available Translated Version | Participants & Settings | N |
| --- | --- | --- | --- | --- | --- |
| SCIRS-SF | English | heterosexual romantic relationships | America and Brazil & Portuguese | Brazilians & heterosexual dating settings | 181 |
| SAVS | English | South Asian immigrant women | Chicago & English | SAI women & most recent relationships | 116 |
| a screen instrument for DV | Persian | Iranian married women | Iran & Persian | married Iranian women & health and social research settings | 334 |
| a single violence question | English | Latina women | Chicago & Spanish or English | Mexican and Puerto-Rican women & community-based healthcare settings | 657 |
| Haj-Yahia's questionnaire | English | female population | Iran & Persian | married women & community-based settings | 471 |
| HARK | English | women in general practice | London & English | women in general practice waiting rooms & clinical settings | 232 |
| NORAQ | English | Arab and Middle Eastern women | Jordan & Arabic | Arab and Middle Eastern women & health -maternal and health care centers | 175 |
| PMWI-SF | English | Portuguese women | Portugal & Portuguese | colleague students & university-based settings | 506 |
| MSDV-2.0 | English | dating relationships | Andalusia & Spanish | university students & educational settings | 1091 |
| IPV-BSS | English | large-scale community populations | Kenya & English | women clients in mental health agencies & clinical and community settings | 8674 |

**Table 5. Methodological Quality and Measurement Property Ratings of Included Instruments**

| Instrument | Structural validity | | | Internal consistence | | | Cross-cultural validity | | | Reliability | | | Criterion validity | | | Hypotheses testing | | | Level of evidence for measurement property | PROM category |
| --- | --- | --- | --- | --- | --- | --- | --- | --- | --- | --- | --- | --- | --- | --- | --- | --- | --- | --- | --- | --- |
|  | n | meth qual | Result (Rating) | n | meth qual | Result (Rating) | n | meth qual | Result  (Rating) | n | meth qual | Result  (Rating) | n | meth qual | Result (Rating) | n | meth qual | Result  (Rating) |  |  |
| IPV-BSS |  |  |  | 544 |  | (+) |  |  |  |  |  |  |  |  |  |  |  |  | High | C |
| SCIRS-SF | 508 |  | (+) | 508 |  | (+) |  |  |  |  |  |  |  |  |  | 508 |  | (-) | Moderate | C |
| SAVS |  |  |  | 116 |  | (+) |  |  |  |  |  |  | 116 |  | (+) |  |  |  | High | C |
| a screen instrument for DV | 334 |  | (+) | 334 |  | (+) |  |  |  | 334 |  | (+) |  |  |  |  |  |  | High | B |
| Haj-Yahia's questionnaire | 471 |  | (+) | 471 |  | (+) |  |  |  | 471 |  | (+) |  |  |  |  |  |  | High | B |
| NORAQ |  |  |  | 171 |  | (+) |  |  |  |  |  |  |  |  |  |  |  |  | High | B |
| PMWI-SF | 506 |  | (-) | 506 |  | (+) |  |  |  | 506 |  | (+) | 506 |  | (+) |  |  |  | Moderate | C |
| MSDV-2.0 | 1091 |  | (?) | 1091 |  | (+) |  |  |  | 1091 |  | (+) |  |  |  |  |  |  | High | B |
| ABI |  |  |  | 400 |  | (+) |  |  |  |  |  |  | 400 |  | (+) |  |  |  | High | C |
| ASIST-GBV |  |  |  | 503 |  | (+) | 503 |  | （+） |  |  |  |  |  |  |  |  |  | High | C |
|  |  |  |  | 998 |  |  | 998 |  |  |  |  |  |  |  |  |  |  |  |  |  |
| WCFSI |  |  |  | 927 |  | (+) |  |  |  |  |  |  |  |  |  |  |  |  | High | C |
| AAT |  |  |  | 205 |  | (+) |  |  |  |  |  |  |  |  |  |  |  |  | Low | C |
| ASI |  |  |  |  |  |  |  |  |  |  |  |  | 699 |  | (?) |  |  |  | Very low | C |
| a single violence question |  |  |  |  |  |  |  |  |  |  |  |  | 657 |  | (?) |  |  |  | Very low | C |
| HARK |  |  |  |  |  |  |  |  |  |  |  |  | 232 |  | (?) |  |  |  | Very low | C |
| PVS |  |  |  |  |  |  |  |  |  |  |  |  | 2461 |  | (?) |  |  |  | Very low | C |
|  |  |  |  |  |  |  |  |  |  |  |  |  | 322 |  |  |  |  |  |  |  |
|  |  |  |  |  |  |  |  |  |  |  |  |  | 116 |  |  |  |  |  |  |  |
| M-PVS |  |  |  |  |  |  |  |  |  |  |  |  | 115 |  | (?) |  |  |  | Very low | C |
| CTS-2 | 209 |  | (-) | 209 |  | (+) |  |  |  |  |  |  |  |  |  |  |  |  | Very low | C |
|  |  |  |  | 768 |  | (+) |  |  |  | 165 |  | (+) |  |  |  |  |  |  |  |  |
|  |  |  |  | 121 |  | (+) |  |  |  |  |  |  |  |  |  |  |  |  |  |  |
|  |  |  |  |  |  |  |  |  |  |  |  |  | 116 |  | (?) |  |  |  |  |  |

very good; adequate; doubtful; inadequate
